# *GRIN3B* missense mutation as an inherited risk factor for schizophrenia: whole-exome sequencing in a family with a familiar history of psychotic disorders

**DOI:** 10.1017/S0016672316000148

**Published:** 2017-01-30

**Authors:** TOBIAS HORNIG, BJÖRN GRÜNING, KOUSIK KUNDU, TORSTEN HOUWAART, ROLF BACKOFEN, KNUT BIBER, CLAUS NORMANN

**Affiliations:** 1 Department of Psychiatry and Psychotherapy, University Medical Center, Albert–Ludwigs University, Hauptstr. 5, 79104 Freiburg, Germany; 2 Bioinformatics Group, Department of Computer Science, University of Freiburg, Georges–Koehler–Allee 106, Freiburg 79110, Germany

## Abstract

Glutamate is the most important excitatory neurotransmitter in the brain. The N-methyl-D-aspartate (NMDA) receptor is a glutamate-gated ionotropic cation channel that is composed of several subunits and modulated by a glycine binding site. Many forms of synaptic plasticity depend on the influx of calcium ions through NMDA receptors, and NMDA receptor dysfunction has been linked to a number of neuropsychiatric disorders, including schizophrenia. Whole-exome sequencing was performed in a family with a strong history of psychotic disorders over three generations. We used an iterative strategy to obtain condense and meaningful variants. In this highly affected family, we found a frameshift mutation (rs10666583) in the *GRIN3B* gene, which codes for the GluN3B subunit of the NMDA receptor in all family members with a psychotic disorder, but not in the healthy relatives. Matsuno *et al.,* also reported this null variant as a risk factor for schizophrenia in 2015. In a broader sample of 22 patients with psychosis, the allele frequency of the rs10666583 mutation variant was increased compared to those of healthy population samples and unaffected relatives. Compared to the 1000 Genomes Project population, we found a significant increase of this variant with a large effect size among patients. The amino acid shift degrades the S1/S2 glycine binding domain of the dominant modulatory GluN3B subunit of the NMDA receptor, which subsequently affects the permeability of the channel pore to calcium ions. A decreased glycine affinity for the GluN3B subunit might cause impaired functional capability of the NMDA receptor and could be an important risk factor for the pathogenesis of psychotic disorders.

## 1. Introduction

The glutamatergic transmission system is the most important excitatory network in the human central nervous system. The ionotropic postsynaptic N-methyl-D-aspartate (NMDA) receptor belongs to the superfamily of glutamate-gated ion channels (Johnson & Ascher, 1987; Dalkara *et al.*, 1992). Three subfamilies (GluN1–3) with seven subunits are currently known (Table 1) (Low & Wee, 2010; Siegel & Brady, 2011; Paoletti *et al.*, 2013). These subunits build heterodimeric or heterotrimeric complexes and form NMDA receptor subtypes and they include different glutamate (GluN2A, B, C and D) and glycine binding domains (GluN1 and GluN3A/B). The glycine binding sites modulate the permeability of the NMDA receptor. As summarized in Table 1, the subunits show differential mRNA expression in the central nervous system (Wee *et al.*, 2008; Low & Wee, 2010; Paoletti *et al.*, 2013).

**Table 1.** N-methyl-D-aspartate receptor subunits, their main transmitter affinity and localization.

Gene family	Subunit	Transmitter affinity	Localization
*GRIN1*	GluN1	Glycine	Ubiquitous
*GRIN2*	GluN2A	Glutamate	FB, CB, MB, HB, PC (layer 5)
	GluN2B	Glutamate	FB, PC (layer 5)
	GluN2C	Glutamate	CB, OB, Hip, cortical interneurons, retina
	GluN2D	Glutamate	Hip, cortical interneurons, retina, MB, HB
*GRIN3*	GluN3A	Glycine	NC (layer 4 and 5), Hip, SC
	GluN3B	Glycine	NC, FB, Hip, Str, CB, SC (motor neurons)

FB: forebrain; CB: cerebellum; MB: midbrain; HB: hindbrain; PC: pyramidal cells; OB: olfactory bulb; Hip: hippocampus; NC: neocortex; SC: spinal cord; Str: striatum.

NMDA receptors are ligand-gated cation channels that are blocked by magnesium ions (Mg^2+^) in the resting state and have functional relevance in learning and memory, synaptogenesis and inducing synaptic plasticity (Harris *et al.*, 1984; Teyler, 1987; Normann *et al.*, 2000; Vargas-Caballero & Robinson, 2004; Normann & Clark, 2005; Fan *et al.*, 2014). Disturbances in NMDA receptor function have been linked to memory and learning deficits and neuropsychiatric disorders such as schizophrenia, major depression and bipolar affective disorders (Zarate *et al.*, 2006; Bitanihirwe *et al.*, 2009; Li *et al.*, 2011; Zhou & Sheng, 2013).

The glutamate hypothesis of schizophrenia was established in the early 1980s by Kim and Kornhuber (Kim *et al*., 1980; Kornhuber *et al*., 1984). In experimental animals, acute blockade of glutamatergic transmission affects the dopaminergic system and induces hyper-dopaminergic conditions in distinct regions of the cerebral cortex (Moghaddam & Adams, 1998; López-Gil *et al.*, 2007). Gene expression and gene polymorphism studies have indicated altered Neuregulin 1 and Dysbindin genes in patients with schizophrenia as causing functional disturbances in glutamatergic synaptic plasticity (Mátyás, 2006; Giegling *et al.*, 2010; Balu & Coyle, 2011; Geddes *et al*., 2011). Moreover, there are indications of abnormal expression of postsynaptic second messenger components of the NMDA receptor, such as the postsynaptic density protein 95 (PSD-95) or glutamate transporters 1 and 2 (Kristiansen *et al.*, 2007; Bauer *et al.*, 2008; de Bartolomeis *et al.*, 2014). Psychopharmacological studies have demonstrated propsychotic effects for glutamate agonists and antipsychotic effects for glutamate antagonists (Javitt, 2009; Blot *et al.*, 2013; Cioffi, 2013; Laruelle, 2014). Unlike the GluN2 subunits that have binding sites for glutamate, the GluN1 and GluN3 subunits have binding sites for glycine. There are suggestions of genetic variations and drug–gene interactions with the *GRIN3B* gene, which encodes GluN3B (Lipsky & Goldman, 2003; Putnam *et al.*, 2011; Tarabeux *et al.*, 2011; Lin *et al.*, 2014). Recently, Matsuno and co-workers found a genetic variation of GluN3B within its coding region (insCGTT) that was significantly overrepresented in patients with schizophrenia, and this was proved to be functionally null (Matsuno *et al.*, 2015).

In 2001, Andersson and colleagues characterized the *GRIN3A* and *GRIN3B* genes (Andersson *et al.*, 2001). The *GRIN3B* gene is localized on chromosome 9q13·3 and consists of eight coding exons. The GluN3B protein, consisting of 1043 amino acids, shows the highest homology with GluN3A and GluN1, and it seems to be a dominant modulatory subunit of the NMDA receptor family (Low & Wee, 2010). The N-terminal extracellular subunit is composed of two lysine/arginine/ornithine-binding protein-like domains (Stern-Bach *et al.*, 1994; Armstrong *et al.*, 1998; Dingledine *et al.*, 1999; Matsuda *et al.*, 2002): S1 (exon 3, amino acid sequence position 422–540) and S2 (exon 4, amino acid sequence position 696–779). These domains have high affinity for glycine (Yao & Mayer, 2006). Interestingly, *GRIN3B*-knockout mice showed deficits in coordination, motor learning and activity, as well as altered social behaviour (Niemann *et al.*, 2007)

Here, we report a family with a strong history of psychotic disorders in which we analysed the exome sequences from eight family members over three generations. We found a missense frameshift mutation in the *GRIN3B* gene in all of the affected family members, but not in any unaffected family member. This mutation variant, which is known as rs10666583, was previously described by Matsuno *et al.* in 2015 as a null variant. Moreover, we show an overrepresentation of the rs10666583 variant in a sample of schizophrenic patients compared to the 1000 Genomes Project database.

## 2. Methods

### (i) Participants

ReelinSys is a systems biology project funded by the German Federal Ministry of Education and Research. In this project, we performed triplet-based exome sequencing in 35 families containing patients with schizophrenia (n = 22) and depression (n = 19) in order to detect *de novo* mutations. In these 35 families, we performed sequencing for both patients and their unaffected parents and siblings. All subjects were of Caucasian ethnicity. Approval was obtained from the ethics committee of Albert–Ludwigs University Freiburg (No. 94/13) before initiation of the study.

**Fig. 1.**
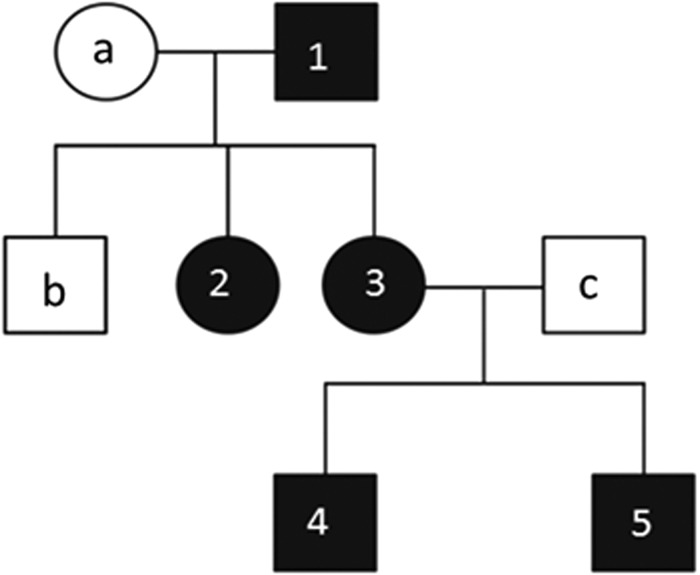
Pedigree of the family, with affected members coloured black. Schizophrenic patients: 1–5; healthy family members: a–c.

During the inclusion process of one of the 22 schizophrenic patients, we registered a history of psychotic disorders in five of their family members over three generations, and only three of their family members had no such history (Fig. 1). We than carried out whole-exome sequencing analysis of all eight of these family members in order to detect all of the mutations that were present only in the affected members. The clinical diagnosis of schizophrenia was made by experienced senior consultant psychiatrists based on a structured interview according to Diagnostic and Statistical Manual of Mental Disorders-5 criteria, and their ability to consent was based on the psychopathological interview according to the Arbeitsgemeinschaft für Methodik und Dokumentation in der Psychiatrie system and refers to the common principles of decision-making. After giving informed written consent to participate on a voluntary basis, the patients were included in this study. We did not include patients who were judicially housed or were situated in legal care.

Patients and their relatives over the age of 18 years were included in this study and all individuals provided written informed consent to participate on a voluntary basis. Because of ethical considerations, one infant sibling was excluded from the study. A medical history was obtained for all patients, and all participants received full neurological and medical examinations, electroencephalogram examinations and routine blood tests in order to exclude individuals with other medical or neurological disorders. The clinical state of psychotic symptoms was assessed using the Positive and Negative Syndrome Scale (Kay *et al.*, 1987), followed by an assessment of formal thought and language disorder (Kircher *et al.*, 2014). The family medical histories and life charts of all family members were documented in electronic case report form. For more details regarding these procedures, refer to the German Clinical Trials Register (DRKS-ID: DRKS00004761). The relevant demographic and clinical data are presented in Table 2.

**Table 2.** Demographic and clinical data of all family members.

Subject	Sex	Age (years)	Diagnosis	Progress	PANSS score	Medication
1	Male	75	Undifferentiated schizophrenia		48	None
2	Female	54	Schizophrenia, paranoid type	Residual	62	None
3	Female	52	Brief psychotic disorder	2 episodes	33	None
4	Male	24	Brief psychotic disorder/seasonal depressive disorder	1 episode	37	Venlafaxine
5	Male	22	Schizophrenia, disorganized type	Residual	72	Aripiprazole
a	Female	73	None		36	None
b	Male	50	None		35	None
c	Male	53	None		39	None

PANSS: Positive and Negative Syndrome Scale.

### (ii) DNA preparation

Blood samples (10 ml) were collected from every subject in ethylenediaminetetraacetic acid tubes. For DNA extraction from peripheral blood leukocytes, we treated the samples using the procedures described in the QIAamp DNA Blood Midi Kit protocol.

### (iii) Gene screening, variation analysis and bioinformatics

All samples were treated with the Agilent SureSelect post-capture and enrichment protocols. Illumina HiSeq 2000 was used to sequence all samples with 100 bp paired end reads.

After quality control measures were performed, all reads were mapped against the human genome build hg19 using Bowtie2. Duplicated reads were removed with the Picard tool suite (version 1·136). We used two different variant calling algorithms (i.e., GATK2·8 and FreeBayes-VERS) for calling variants (McKenna *et al.*, 2010; Garrison & Marth, 2012). For both calling algorithms, we followed the reported best practices and guidelines. Functional annotation of genetic variants was performed with SnpEff version 3·6 (Cingolani *et al.*, 2012). Finally, all annotated variants were loaded into a GEMINI database, and these were further used to explore the forms of human genetic variations (Paila *et al.*, 2013).

We used an iterative strategy to obtain condense and meaningful variants. The strategy included the following steps: (i) quality of the reads; (ii) read depths (DP); (iii) exon-specific mutations; (iv) novel variants that were not present in the dbSNP database, etc.; and (v) comparison of our VCF files with the 1000 Genomes Project database (1000 Genomes Project Consortium, 2012), testing the significance with RStudio (version 7·7) Fisher's exact test.

#### (a) Tertiary and quaternary protein modelling

We built SWISS-MODEL tertiary and quaternary protein models using amino acid sequences from the National Center for Biotechnology Information (NCBI) database (FASTA format) and using a template with 25% (wild-type) and 18% (rs100666583 variation) sequence identities, respectively. For these models, the coverage was high. The SWISS-MODEL is an automated, non-profit, protein homology modelling server created by the Biozentrum at the University of Basel using evolutionary information (Schwede *et al.*, 2003; Biasini *et al.*, 2014).

## 3. Results

### (i) Identification of disorder-specific mutations

Exome sequencing was performed for all family members (Fig. 1). We found a non-synonymous mutation that satisfied the high-impact quality demands (see ‘Gene screening, variation analysis and bioinformatics’ section), and it was detected only in the patients with psychotic disorders.

#### (a) Missense mutation in the *GRIN3B* gene

**Fig. 2.**
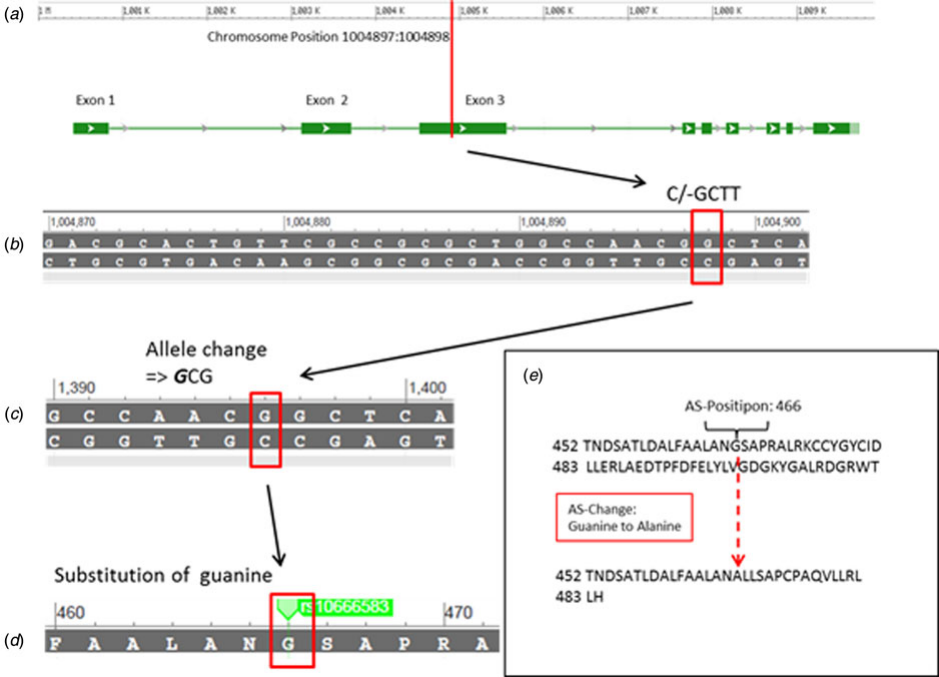
The *GRIN3B* rs10666583 mutation causes an altered amino acid structure. The *GRIN3B* rs10666583 mutation (box) on chromosome 19 in exon 3 [44] (*a*) requires an insertion variation –/GCTT (*b*) that causes an allele change on the mRNA strand (*c*), which lead to an amino acid change from guanine to alanine and an altered AAS of GluN3B (*d*) starting from position 466 (*e*).

We found a missense mutation in the S1 domain of the GluN3B subunit, which was a single-nucleotide polymorphism on chromosome 19 position 1004897:1004898. This mutation is known as rs10666583 and is listed in the NCBI database dbSNP. The –/CGTT insertion causes an mRNA allele change and an amino acid residue frameshift. As a consequence of the mutation, guanine is replaced by alanine and an amino acid sequence is altered at position 466 within the glycine binding side of the S1 domain. The modified amino acid sequences are shown in Fig. 2.

#### (b) rs10666583 in the schizophrenic and healthy population of ReelinSys

Over the course of the ReelinSys project, we analysed the exome sequences of 17 out of 35 families containing at least one or more persons with a psychotic disorder, leading to a total of 22 patients. We found the rs10666583 mutation variant in 15 alleles, with an allele frequency (AF) of 0·51 in a total of 14 patients. Among the 34 corresponding healthy parents in the ReelinSys project, we found that 14 possessed this mutation, with an AF of 0·25. We did not find spontaneous rs10666583 mutations in any of the schizophrenic mutation carriers. This means there was no significant increase of the rs10666583 AF in patients compared with the healthy parents (*p* = 0·12).

### (ii) rs10666583 in the schizophrenic patients compared with the 1000 Genomes Project population

We compared the genotype frequency (GF) – the quotient of individuals with a given genotype and the total number of individuals in a population – of rs10666583 in our ReelinSys study population with the 1000 Genomes Project database population (Table 3). We found that 14 out of 22 patients affected by psychotic disorder had this *GRIN3B* variant (GF = 0·63). From the 1000 Genomes Project population, 719 out of 2504 individuals had the rs10666583 variant (GF = 0·28). This represents a significant increase among patients (*p* = 0·0013), with an odds ratio of 4·3 (95% confidence interval [CI]: 1·8–10·4). We found 797 alleles with this variant out of 4211 in the 1000 Genomes Project population, with a AF of 0·18, and this represents a significant increase of rs10666583 in patients (*p* = 0·0018), with an odds ratio of 2·2 (95% CI: 1·2–4·3).

**Table 3.** rs10666583 variant mutation carriers.

WP/AF	WP/GF	SZ/AF	SZ/GF	Sib/AF	SMR
0·18	0·28	0·51^*a*^	0·63^*a*^	0·25	None

a Significant increase (*p* < 0·05) of GF and AF in patients compared to WP.

WP: world population; AF: allele frequency; GF: genotype frequency; SZ: schizophrenic patients; Sib: siblings; SMR: spontaneous mutation rate.

### (iii) SWISS protein model of the GluN3B wild-type and rs10666583 mutation variant

**Fig. 3.**
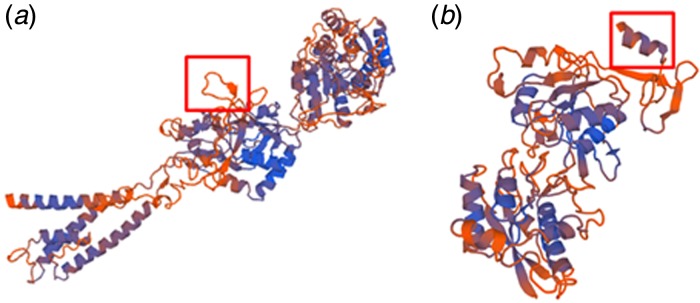
The SWISS model of wild-type Glu3B and the rs10666583 variation. The SWISS protein model shows the wild-type Glu3B subunit (*a*) of the N-methyl-D-aspartate receptor; the S1 region is boxed. The rs10666583 variation (*b*) shows truncated and altered protein tertiary and quaternary structures with the loss of its S1 region.

The 3D-SWISS protein model is shown in Fig. 3. As Fig. 3 indicates, the level of certainty of the structure ranges from high (blue) to low (orange). Because of the amino acid change and the frameshift of the amino acid structure at position 466, the model shows altered tertiary and quaternary structures for this region (Fig. 3(*b*), boxed region). The rs10666583 variant stops at position 482, and hence is significantly shorter than the wild-type GluN3B subunit, which is 1043 amino acids long.

## 4. Discussion

Our main finding was of an autosomal dominant heterozygous frameshift mutation in the *GRIN3B* gene encoding the GluN3B subunit of the NMDA receptor that was present in all of the affected family members of our pedigree analysis and in 63·6% of all patients with schizophrenia in our ReelinSys study population. Compared to the 1000 Genomes Project database, we found a significantly increase in the quantity of this *GRIN3B* variant.

This frameshift causes an amino acid residue change at position 466 in the GluN3B subunit, which implies a displacement of the S1 binding domain primary structure that could reduce the affinity for glycine. A mutation analysis of the mouse GluN3B S1 domain and the critical amino acid residue sequences at positions 424, 504 and 505 revealed a distinct loss of the glycine binding site (Awobuluyi *et al.*, 2007). Furthermore, we demonstrated altered tertiary and quaternary structures of the glycine binding site in the S1 region in our 3D-SWISS-MODEL; in addition, the mutant protein was significantly shorter than the wild-type protein. These findings are consistent with the subcellular findings of Matsuno and co-workers, who also described a truncated molecule with a lighter molecular weight and smaller bands than the major wild-type protein (Matsuno *et al.*, 2015). Additionally, these authors found that the NMDA receptor rs10666583 mutation variant of the GluN3B subunit was not functioning upon electrophysiological verification (Matsuno *et al.*, 2015). Glycine binding in the S1/S2 domain of GluN3B mediates the properties of the receptor pore. The GluN3/GluN2 and GluN1 heterotrimer leads to a reduction in Ca^2+^ permeability for the corresponding NMDA receptor, whereas the heterodimer GluN3/GluN1 leads to an increase in Ca^2+^ permeability (Chatterton *et al.*, 2002; Matsuda *et al.*, 2002, 2003; Low & Wee, 2010). Functional disturbances in these modulatory mechanisms could therefore lead to insufficient regulation of dendritic, spine or synaptic plasticity, or even cell death, induced by Ca^2+^ and a subsequent desynchronization of cortical microcircuits (Yuan *et al.*, 2003; Alvarez & Sabatini, 2007; Zhang *et al.*, 2007; Konstantoudaki *et al.*, 2014). Interestingly, Spiros and colleagues found an inverse U-shaped dose–response correlation between glycine concentration and the negative symptoms of schizophrenia as a potential consequence of an imbalance in the ratio between excitation and inhibition (Spiros *et al.*, 2014). Post-mortem histological analyses of cerebral brain tissue in patients with schizophrenia indicated a reduced pyramidal cell density and dendritic spine alterations in several regions of the cerebral cortex (Harrison, 1999; Benes *et al.*, 1986; Glausier & Lewis, 2013). The core symptoms of psychotic disorders, which include hallucinations, delusions and cognitive dysfunctions, are most likely due to dysfunctional neuronal circuits, unbalanced neurochemistry and reduced neuronal plasticity, and could therefore be linked to deregulated NMDA receptor functioning.

The rs10666583 frameshift was observed in 18% (AF) of the global population according to the 1000 Genomes Project, and in 25% (AF) of the healthy relatives in our study population. With a prevalence of psychotic disorders of less than 1% in the total population, these findings suggest that this polymorphism is not the only cause of the disease. Such a finding could also explain the increased rate of mutation carriers among the healthy relatives in the present study populations. We found distinctly higher rs10666583 AF and GF values among the patients in our population compared to the 1000 Genomes Project database. With odds ratios of 4·3 for the GF and 2·2 for the AF, this finding highlights the potential impact of this variant in the pathogenesis of schizophrenia, and we point out that a false allocation of pathogenicity could have severe consequences for patients and research enterprises.

Interestingly, we found rs10666583 only as a hereditary mutation and not as a *de novo* mutation. Referring to Matsuno *et al*. (2015), we also demonstrated that this variant occurred within a family only for affected members, and that this variation is overrepresented in patients with psychotic disorder compared to in the 1000 Genomes Project. Because of the unbiased replication of this dbSNP based on undirected whole-exome sequencing, this provides added value beyond the specific genotyping of Matsuno *et al*. (2015). In addition, an inherited mutation variant, which occurs only in diseased family members, represents a stronger indication of the development of the disease than a case–control study in the general population. It is precisely this biological importance that adds value to previous data, as it is a random finding.

An important limitation of this study is that, as the 1000 Genomes Project cannot rule out the future onset of a psychiatric disorder in previously healthy participants, it seems unlikely that all mutation carriers will eventually develop a psychiatric disease. Unknown protective genes and other modulating mechanisms may therefore be postulated in healthy mutation carriers. At the time of this writing, there were no genomic databases focusing on genetic variation among patients with psychiatric disorders and their siblings with which we could compare our results.

## 5. Conclusion

We found a frameshift mutation in the GluN3B subunit of the NMDA receptor that induces a degradation of the S1 glycine binding domain. This mutation could play a role in the deregulated neuronal circuits and neuronal plasticity in patients with psychotic disorders. Because of the high prevalence of this dbSNP in the non-affected population, we suggest that there is a complex interaction of further polymorphisms that causes the manifestation of psychotic symptoms. An important task for the future is to build a genomic database for psychiatric disorders in order to re-evaluate detected polymorphisms in patients with psychiatric disorders and to uncover the polygenetic causes of such disorders.

## 9. Availability of data and materials

After acceptance of BPSY-D-16-00377, a DNA database including a genomic variants archive will be set up online.
